# Retraction: Aryl glyoxal: a prime synthetic equivalent for multicomponent reactions in the designing of oxygen heterocycles

**DOI:** 10.1039/d5ra90032h

**Published:** 2025-04-30

**Authors:** Abdur Rehman Sheikh, Anam Arif, Md. Musawwer Khan

**Affiliations:** a Department of Chemistry, Aligarh Muslim University Aligarh 202002 India musawwer@gmail.com

## Abstract

Retraction of ‘Aryl glyoxal: a prime synthetic equivalent for multicomponent reactions in the designing of oxygen heterocycles’ by Abdur Rehman Sheikh *et al.*, *RSC Adv.*, 2023, **13**, 11652–11684, https://doi.org/10.1039/D2RA08315A.

We, the named authors, hereby wholly retract this *RSC Advances* article due to a number of typographical errors, errors in the referencing, and errors in the naming and structures of compounds. Due to the number of errors, this version of record is not accurate. The original article will therefore be retracted.

Having consulted with independent experts, the Royal Society of Chemistry has determined that any changes made to the paper to correct this would be major, and therefore the best course of action is retraction and republication of the article with all errors addressed. The Royal Society of Chemistry is happy that the overall conclusions of the paper are not affected by these errors, and therefore that republication of the work with the correct information is appropriate. The republished article was peer reviewed and can be found at [https://doi.org/10.1039/D5RA01953B].

The corresponding authors regret this oversight and apologise for any inconvenience to readers.

Signed: Abdur Rehman Sheikh, Anam Arif and Md. Musawwer Khan

Date: 24th March 2025

Retraction endorsed by Laura Fisher, Executive Editor, *RSC Advances*

